# The Influence of Diabetes Mellitus in Myocardial Ischemic Preconditioning

**DOI:** 10.1155/2016/8963403

**Published:** 2016-08-30

**Authors:** Paulo Cury Rezende, Rosa Maria Rahmi, Whady Hueb

**Affiliations:** Department of Atherosclerosis, Heart Institute of the University of São Paulo Medical School, São Paulo, SP, Brazil

## Abstract

Ischemic preconditioning (IP) is a powerful mechanism of protection discovered in the heart in which ischemia paradoxically protects the myocardium against other ischemic insults. Many factors such as diseases and medications may influence IP expression. Although diabetes poses higher cardiovascular risk, the physiopathology underlying this condition is uncertain. Moreover, although diabetes is believed to alter intracellular pathways related to myocardial protective mechanisms, it is still controversial whether diabetes may interfere with ischemic preconditioning and whether this might influence clinical outcomes. This review article looks at published reports with animal models and humans that tried to evaluate the possible influence of diabetes in myocardial ischemic preconditioning.

## 1. Ischemic Preconditioning

After the seminal work of Murry and coworkers in 1986 [1] who were the first to demonstrate that brief ischemia may make the myocardium more resistant to a severe ischemic insult [1], many experimental studies have tried to explain this cardioprotective phenomenon called ischemic preconditioning (IP). The great protection observed in experimental models, which has shown a reduction of 75% in the infarcted area [1] compared with that in animals not exposed to the phenomenon, has caught the attention of the scientific community. Efforts are, therefore, being made to try to understand the mechanisms of IP and translate them into clinical benefits. IP is considered the most intense cardioprotective phenomenon discovered so far.

After IP was identified, its cardioprotective benefit was at first thought to be a result of a vasodilatory phenomenon that occurred in the coronary arteries after the initial ischemic insult [2]. However, studies have shown that IP occurs irrespective of the contribution of collateral vessels or higher coronary flow [3, 4]. Currently, IP is understood to be a complex intracellular mechanism, with many and redundant metabolic pathways, which make the myocardial cell temporarily more resistant to a severe and prolonged ischemic insult [5, 6].

In the last 30 years many authors have tried to understand the mechanisms responsible for cardioprotection and much has been discovered [6]. Currently it is known that reperfusion is a fundamental part of the preconditioning stimulus, and after the first cycles of brief ischemia and reperfusion, some substances are produced and released by the cardiomyocytes such as adenosine, bradykinin, endothelin, opioids, and acetylcholine. They bind to specific receptors on the membrane of the cardiomyocytes and activate pathways in the mitochondria, leading to the activation of protein kinases by reactive oxygen species. These protein kinases such as Akt, Erk1/2, and protein kinase C result in the recruitment of major pathways such as the Reperfusion Injury Salvage Kinase (RISK) pathway, the Survivor Activator Factor Enhancement (SAFE) pathway, and the cGMP-protein kinase G (PKG) pathway. These salvage pathways activate downstream mediators such as endothelial nitric oxide synthase (eNOS), protein kinase C*ε* (PKC*ε*), and the mitochondrial ATP-dependent potassium channel (K-ATP channel), which have an inhibitory effect on mitochondrial permeability/transition pore (MPTP) opening. It has been shown that the inhibition of the MPTP opening contributes to cardioprotective effects [6–8].

Moreover, other studies have confirmed that potassium ATP channels both in the cell membrane and in mitochondria play a fundamental role in IP triggering [9]. And once triggered, complex mechanisms are initiated and finally shift the metabolism of the inner cells into a more ischemia-resistant state. Based on these discoveries, some works demonstrated the action of many medications and diseases in these pathways blocking or stimulating the specific cell machinery [10, 11]. Most notably, many of these studies have shown that some specific classes of medications, such as antidiabetic agents, and especially those that can block K-ATP channels in the myocardium may block ischemic preconditioning [12]. Furthermore, it has also been suggested that diabetes per se may interfere with these channels [13] and other IP pathways [14] and then compromise the triggering of this myocardial protective phenomenon.

## 2. Clinical Significance of Ischemic Preconditioning

Many centuries before the discovery of the experimental phenomenon called IP and, actually, during the first observations of patients with angina symptoms, some notable physicians such as Heberden [15] and Osler [16] observed that some patients with angina had a very peculiar and characteristic symptom: warm-up and walk-through angina. Patients with such symptoms described angina that had improved or even disappeared with the continuation of exercise or with a brief rest followed by the restart of exercise. Interestingly, these observations may still be made by some coronary artery disease patients, especially if they are carefully questioned about these angina characteristics.

The clinical observation of warm-up angina was further analyzed by electrocardiographic studies [17, 18]. These studies show that the improvement in symptoms was followed by improvement in ischemic electrocardiographic parameters. This observation allowed the application of sequential exercise testing to observe the assumed clinical manifestation in humans afforded by IP [18]. Moreover, based on these previous reports and other invasive studies [3, 4], it is currently assumed that warm-up and walk-through angina may be clinical manifestations of the experimental phenomenon called IP.

After the discovery of some of the pathways related to IP, many studies have demonstrated the impact of medications on IP, both in experimental and in human studies [19]. So, because antidiabetic drugs block K-ATP channels [9], many authors have described the negative interference of these drugs with IP [12], especially glibenclamide, a sulfonylurea that causes blockage of the K-ATP channels in the pancreas causing the secretion of insulin but that may also act in these channels in the heart, possibly causing the blockage of IP [20]. This is one of the possible explanations for the occurrence of more cardiovascular outcomes in the patients with diabetes hospitalized due to an acute coronary syndrome and who were being treated with such drugs [21].

Actually, few studies have tested the clinical protection of IP in terms of clinical outcomes. One such study was conducted by Ishihara and coworkers [22]. They evaluated whether preinfarct angina is associated with fewer clinical endpoints in patients hospitalized due to an acute myocardial infarction. In this retrospective study, patients who had an acute myocardial infarction were assessed in terms of the presence of preinfarct angina that was assumed to be any thoracic pain within 24 hours prior to the infarct episode. In such patients, the authors evaluated the release of markers of myocardial necrosis, ventricular dysfunction, and hospital mortality and compared these results between patients with and without preinfarct angina. The authors showed in the group of patients without diabetes that those who had preinfarct angina had a lower release of markers of myocardial necrosis, better recovery of ventricular function, and lower rates of hospital mortality. On the other hand, this study showed that, in the population of patients with diabetes, patients with preinfarct angina had similar outcomes compared to the group of patients with no preinfarct angina. Thus, this study showed that IP represented by preinfarct angina may be associated with better clinical outcomes and that diabetes may compromise this cardioprotective phenomenon. However, despite these results, it is still uncertain whether IP may be associated with better clinical outcomes.

## 3. Diabetes Mellitus and Ischemic Preconditioning

Patients with both diabetes and ischemic heart disease are supposed to have more aggressive coronary artery disease and poorer cardiovascular outcomes [23, 24]. However, this is controversial, because of the great variability in disease states regarding diabetes and its association with other cardiovascular risk factors. Moreover, the causes of this supposed worse prognosis are not well understood and impairment in myocardial protective mechanisms is supposed to be one of the possible explanations [25, 26].

Although it has been tested in many animal and human studies, the relation between diabetes mellitus and myocardial ischemic preconditioning is a matter of intense debate and controversy [27].

Although some IP pathways are believed to be possible targets of diabetic metabolic alterations, the results of many experimental studies are conflicting [27]. Some studies have demonstrated that diabetes may impair IP [25, 26]; however, others suggest that this mechanism is preserved in diabetic models [28, 29].

This great variability in results is probably due to important methodological differences. The animal models are quite different among studies, as well as the protocols to induce diabetes, the duration of the disease, and the endpoints that are considered in terms of cardioprotection. Thus, there are studies with dogs, rats, and rabbits that show different responses in terms of cardioprotection [28, 30, 31]. On the other hand, experimental models examining a short period in the diabetic state are more prone to preserve cardioprotective phenomenon, while those with long periods of disease frequently show the loss of this protection [32]. Moreover, some studies have assessed cardioprotection by the analysis of the infarcted myocardial area, while others evaluated the recovery of ventricular function, and others studied the occurrence of ventricular arrhythmias. It is important to emphasize that each of these biological variables represents distinct pathophysiologies and may be diversely affected by ischemic preconditioning.

Although they are not completely understood, many IP-related pathways have been suspected as possible targets of diabetes impairment [7]. Studies have shown that diabetes may alter both sarcolemmal and mitochondrial K-ATP channels and then alter mitochondrial function [25]. This diabetes-related dysfunctional mitochondria might lead to an elevated superoxide production and, thus, to a higher susceptibility to cellular injuries [25]. Other signaling pathways have also been shown to be altered by diabetes. Some studies have shown that diabetes may impair the activation of PI3K-Akt-eNOS signaling, as well as some upstream mechanisms and this was shown to be related to decreased generation of nitric oxide and attenuated effect of IP-mediated cardioprotection [8]. It has been suggested that hyperglycemia may be responsible for this altered function because it may impair nitric oxide action [33] and afterwards impair the action of K-ATP channels openers in cardiac cells [34].

A reduced phosphorylation of ERK1/2 was also observed in chronically diabetic animal models and this was associated with greater myocardial infarct size [35]. Interestingly, some other authors have shown a reduced release of calcitonin gene-related peptide (CGRP) in diabetic animal models and an attenuation of IP-induced cardioprotection [36]. Taken together these studies show that many IP pathways as well as mitochondrial channels and function may be disrupted by diabetes and impose an altered cardiac response to ischemic injuries. Interestingly, these findings were demonstrated in chronically experimental diabetic myocardium, while acute and subacute models showed distinct responses [7].

Human studies that have tried to evaluate the possible interference of diabetes with ischemic preconditioning are scarce. Some of them have studied myocardial human tissue responses [37, 38] and others have assessed clinical endpoints [22, 39].

Ghosh and coworkers [37] have studied in vitro myocardial damage of human atrial appendages from patients with and without diabetes. The authors assessed the release of markers of myocardial necrosis and the percentage of tissue viability after a severe ischemic insult in patients with diabetes receiving dietary treatment only, in those taking insulin, and in those taking oral hypoglycemic drugs and compared them with those in patients without diabetes. All the groups underwent ischemic or pharmacologic preconditioning. Among other findings, they demonstrated a similar intensity of cardioprotection between patients with and without diabetes, although they found lower protection in patients with diabetes who were taking insulin or oral antidiabetic medications. Thus, in this study, diabetes did not interfere with the cardioprotection afforded by IP.

Another study by Cleveland Jr. and coworkers [38] assessed the contractile strength of isolated trabeculae of right atrial appendages from patients with chronic coronary artery disease, resected during bypass surgery. They assessed the improvement in the contractile strength after a prolonged ischemic insult followed by reperfusion in myocardial tissue from patients with diabetes taking insulin, those taking oral antidiabetic drugs (glibenclamide or glipizide), and patients without diabetes. All the groups had experienced a previous ischemic insult. The authors showed that the group of diabetic tissues had a similar improvement in the contractile strength compared to nondiabetic tissues and that the groups treated with oral hypoglycemic drugs had a lower recovery of contractile function.

**Figure 1:**
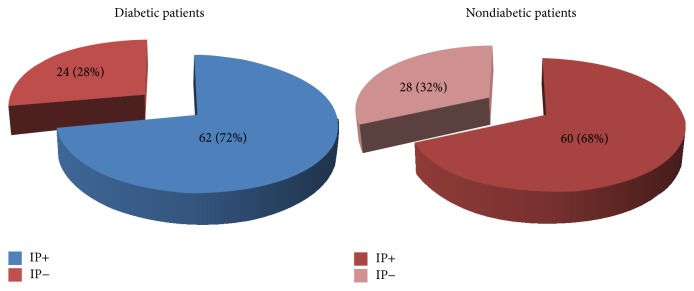
Pie charts showing the number and percentage of patients with and without diabetes who demonstrated ischemic preconditioning (IP). Extracted from [40].

**Figure 2:**
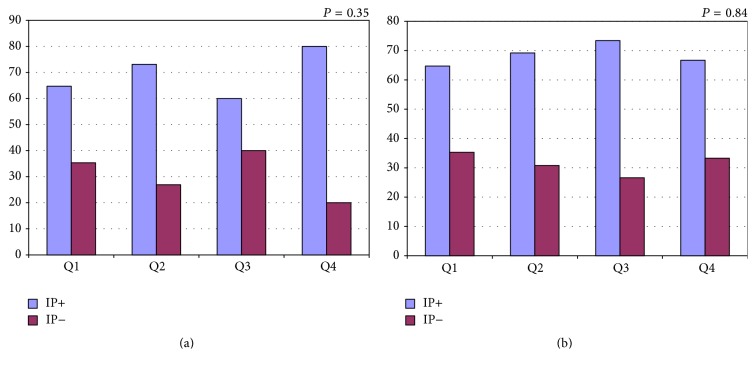
Graphs showing the percentage of patients who demonstrated ischemic preconditioning (IP+ in blue) and who did not demonstrate ischemic preconditioning (IP− in red) stratified into quartiles of A1c (a) and Fasting Glycemia (b). IP = ischemic preconditioning; Q = quartile (s). *x*-axis represents the percentage of patients, and *y*-axis represents the quartiles of A1c and Fasting Glycemia. Extracted from [40].

A study from our research group evaluated ischemic preconditioning by sequential treadmill exercise stress testing in 174 patients with coronary artery disease and with and without diabetes [40]. We assessed the improvement in ischemic parameters as the time to reach 1.0 mm ST-segment deviation (T-1.0 mm) and rate-pressure product and compared the results between 2 sequential tests. Besides diabetes status, both groups were well balanced regarding most demographic variables. Contrary to the initial expectation, we found that, among the 86 patients with diabetes, 62 (72%) had an improvement in T-1.0 mm consistent with IP and that among the 88 patients without diabetes, 60 (68%) had an ischemic improvement consistent with IP (Figure 1, *P* = 0.62) [40]. Interestingly, our study also showed in the diabetic population a better result in terms of rate-pressure product, which is a variable that represents myocardial oxygen consumption. One major question is that the population with diabetes was receiving well-controlled treatment for diabetes, which can be noted by the controlled results of glycated hemoglobin. Yet, different results could be found in other populations with different diabetic statuses. On the other hand, even when patients were stratified according to their glycated hemoglobin, the higher quartiles were not associated with lower incidences of myocardial protection (Figure 2) [40]. Although this result confirms the results of studies with human myocardial tissue, it contradicts the results of 2 other studies with human populations [22, 39].

Lee and Chou [39] studied coronary artery disease patients with and without diabetes during percutaneous intervention and assessed the action of hypoglycemic agents on IP. Thus, patients with angina symptoms, positive stress testing, and coronary lesions indicated for percutaneous treatment underwent sequential coronary balloon inflations. During these inflations, the authors evaluated angina intensity, ST-segment deviation by intracoronary electrocardiography, and lactate production by measures from the venous cardiac sinus. The authors observed that the group of patients participating in the IP protocol (brief ischemic coronary inflations) had a lower intensity of angina symptoms, lower lactate levels, and lower ST-segment deviation. They also noted that the group of patients with diabetes treated with glimepiride had higher lactate production compared with patients without diabetes treated with glimepiride. Aside from this result, a major limitation of this study is that there was not a direct comparison between IP from patients with and without diabetes, with no action of hypoglycemic agents.

Ishihara et al.'s study has been discussed previously in terms of the clinical endpoints in the population without diabetes [22]. Here, we discuss the results of the population from this study with diabetes. Thus, the authors have assessed the effects of preinfarct angina in the peak of the markers of myocardial necrosis, ventricular function, and hospital mortality, in patients with and without diabetes, hospitalized due to an acute myocardial infarction. In patients without diabetes who had preinfarct angina, the authors found lower peaks of biomarkers of necrosis, better recovery of ventricular function, and lower hospital mortality. However, in the group of patients with diabetes, they did not find any differences in these parameters when they compared patients with or without preinfarct angina. However, although the authors have concluded that the presence of diabetes may prevent the occurrence of ischemic preconditioning, many issues limit these conclusions. This was a retrospective study, in which the number of patients with diabetes was too small (*n* = 121), especially compared with the group of patients without diabetes (*n* = 490). In addition, 53 of the 121 patients with diabetes were being treated with hypoglycemic agents, and many studies have shown that some frequently used hypoglycemic agents may block IP [12, 20].

Thus, both studies that suggest that IP may be impaired by diabetes mellitus have important methodological limitations.

Another study by Bilinska and coworkers [41] had patients with coronary artery disease and with and without diabetes undergo sequential bicycle exercise tests. Although this study had a smaller number of patients with diabetes and the major evaluation was to test antidiabetic drugs, the comparison of patients with diabetes on oral dietary treatment shows similar results compared with patients without diabetes. These results are concordant with the findings of our research center.

Although the information from experimental studies is valuable, the translation of such results to humans is complicated, especially concerning diabetic metabolic alterations. In humans, the association of diabetes with other metabolic dysfunctions and other cardiovascular risk factors results in interaction between them and may result in more aggressive disease. In animal models, the majority of studies cannot associate other metabolic states and diseases, or other risk factors, frequently found in human populations with diabetes. Regarding human studies, they are scarce and the few ones performed so far have contradictory results. It is possible that most of these differences may be attributable to the interference of antidiabetic medications or to differences in the effectiveness of glycemic control in short as well as in chronic states. Assuming that hyperglycemia may negatively influence protective mechanisms [42], it is possible that the control of metabolic changes may restore intracellular signaling protective mechanisms [27] and influence the findings of these studies.

Finally, the influence of the diabetic state in IP mechanisms will need further study to better comprehend their interdependence and to find effective ways to preserve cardioprotective effects and hopefully improve major cardiovascular outcomes.
